# The involvement of anti-inflammatory protein, Annexin A1, in ocular toxoplasmosis

**Published:** 2012-06-15

**Authors:** Kallyne K. Mimura, Roberto C. Tedesco, Katia S. Calabrese, Cristiane D. Gil, Sonia M. Oliani

**Affiliations:** 1 From the Post-Graduation Structural and Functional Biology, Federal University of São Paulo (UNIFESP), São Paulo, SP, Brazil; 2 Department of Morphology and Genetics, Federal University of São Paulo (UNIFESP), São Paulo, SP, Brazil; 3 Laboratório de Imunomodulação e Protozoologia, Instituto Oswaldo Cruz/FIOCRUZ, Rio de Janeiro, RJ, Brazil; 4 Department of Biology, Instituto de Biociências, Letras e Ciências Exatas (IBILCE), São Paulo State University (UNESP), São José do Rio Preto, SP, Brazil

## Abstract

**Purpose:**

The aim of this study was to evaluate the expression of the protein annexin A1 (ANXA1), a potent endogenous regulator of the inflammatory process, in ocular toxoplasmosis.

**Methods:**

C57BL/6 female mice were infected using intravitreal injections of either 10^6^ tachyzoites of *Toxoplasma gondii* (RH strain; *T. gondii*) or PBS only (control groups). After 24, 48, and 72 h, animals were sacrificed and their eyes were harvested for histopathological, immunohistochemical, and ultrastructural immunocytochemical analysis of ANXA1. Human retinal pigment epithelial (RPE) cells (ARPE-19) were infected in vitro with *T. gondii* and collected after 60, 120, 240 min, and 24 h.

**Results:**

Compared with non-infected eyes, an intense inflammatory response was observed in the anterior (24 h after infection) and posterior segments (72 h after infection) of the infected eye, characterized by neutrophil infiltration and by the presence of tachyzoites and their consequent destruction along with disorganization of normal retina architecture and RPE vacuolization. *T. gondii* infection was associated with a significant increase of ANXA1 expression in the neutrophils at 24, 48, and 72 h, and in the RPE at 48 and 72 h. In vitro studies confirmed an upregulation of ANXA1 levels in RPE cells, after 60 and 120 min of infection with *T. gondii*.

**Conclusions:**

The positive modulation of endogenous ANXA1 in the inflammatory and RPE cells during *T. gondii* infection suggests that this protein may serve as a therapeutic target in ocular toxoplasmosis.

## Introduction

Retinochoroiditis is the most common manifestation of ocular toxoplasmosis and is often the only clinical symptom of congenital toxoplasmosis [1,2]. This condition can also induce inflammation in the iris, ciliary body and cataracts [3]. In active ocular toxoplasmosis the choroid exhibits vascular changes, hemorrhage, inflammatory infiltrates, and edema, which may cause optic neuritis [4,5].

Experimental assays using animals to investigate the effects of *Toxoplasma gondii* (*T. gondii*) in the eye have been important for a better understanding of the pathogenesis of human ocular toxoplasmosis. The macrophages, lymphocytes, plasma cells, and mast cells that infiltrate infectious lesions, are detected mainly around blood vessels, and release potent inflammatory mediators, including histamine, in particular, which contributes to ocular inflammatory processes [6]. Mast cells are highly specialized cells that synthesize and secrete a variety of pharmacologically active products present in their cytoplasmic granules, including acid phosphatase, peroxidase, beta-glucuronidase, RNase, DNase, and anionic proteins that give their classical staining patterns [7]. After mast cell degranulation, these associated factors can trigger chemotaxis and the transmigration of leukocytes to the sites of tissue injury [8].

In addition to inflammatory cells, the retinal pigment epithelium (RPE) plays important roles in the inflammatory, immune and healing processes during ocular inflammation in the immunosuppression of lymphocyte proliferation and activation of macrophages by prostaglandin production and transforming growth factor beta (TGF-β) secretion, respectively [9].

Recent studies have elucidated that in addition to cellular activation induced by pharmacological mediators and pro-inflammatory pathways during the parasitic infection, the immune response is also partially attributed to other systems that modulate the inflammatory events. One of the endogenous inflammatory mediators that is induced by glucocorticoids and that is responsible for the inhibition of eicosanoid and phospholipase A2 synthesis is the annexin A1 protein (ANXA1) [10]. In addition to its anti-inflammatory activities, ANXA1 is involved in other pathways of inflammation, such as the potentiation of apoptosis [11,12] and the inhibition of necrosis induced by hydrogen peroxide in rat thymocytes [13].

The expression of ANXA1 was detected in several tissues and cell types, including neutrophils [14,15], monocytes [16], mast cells [17,18], and epithelial cells [19]. However, the increased expression of this protein may occur during a systemic inflammatory reaction, such as that observed in an experimental model of lipopolysaccharide (LPS)-induced endotoxaemia [20]. This model demonstrated that ANXA1 expression depends on the combined actions of endogenous glucocorticoids, interleukin 6 (IL-6) and tumor necrosis factor-α (TNF-α). Furthermore, studies demonstrated that the ANXA1 protein critically regulates acute and systemic inflammation induced by zymosan and LPS, respectively [21,22].

Despite these findings, the role of ANXA1 in the eye remains relatively unstudied. This study evaluated the expression of ANXA1 in the ocular tissues of mice intravitreally infected with *T. gondii* (RH strain) and investigated parasite/host cells interactions in vitro using ARPE-19 cells.

## Methods

### Animals

Four- to six-week-old female C57BL/6 mice (bodyweight 15 to 18 g) were obtained from the animal facilities of the Oswaldo Cruz Foundation, Rio de Janeiro, Brazil. The animals were fed commercial chow and water ad libitum, and were maintained in a 12 h light/dark cycle. All experiments were performed in accordance with the guidelines for experimental procedures of Oswaldo Cruz Foundation (process number L.0228/04).

### Parasites

*T. gondii* tachyzoites of the RH strain were maintained by serial passage in female Swiss mice. For experimental infections, tachyzoites were obtained from the peritoneal exudates of mice, which were washed twice in1640 RPMI (Sigma, St. Louis, MO) containing 50 mg/l gentamicin. The viable parasites were counted in a Neubauer hemocytometer chamber in suspensions containing trypan blue.

### Experimental design

Animals were divided into 2 groups of 12 animals each and were inoculated by intravitreal injection of 10^6^ tachyzoites in 5 µl of PBS (experimental group) or by 5 µl of PBS alone (control group) [23]. Four animals from each group were killed at 24, 48, and 72 h post-infection in a CO_2_ chamber. The eyes were enucleated, fixed and processed for histopathological, immunohistochemical and immunocytochemical analysis as described below.

### ARPE-19 culture conditions

Retinal pigment cells (ARPE-19), isolated from a human eye in 1986 [24] were purchased from ATCC (register number CRL2302; Manassas, VA). ARPE-19 cells were maintained in 25 cm^2^ tissue culture flasks containing 5 ml of Dullbecco’s Modified Eagle’s medium and Ham F-12 (DMEM-F12; D5523; Sigma-Aldrich, Dublin, Ireland), supplemented with 10% fetal calf serum (SFB; CULTILAB, Campinas, SP, BR), 200 mM L-glutamine (G5792; Sigma-Aldrich) and 100 U/ml of penicillin (P7794; Sigma-Aldrich), and incubated at 37 °C under 5% of atmosphere CO_2_.

### ARPE-19 cell infection of *T. gondii*

For the infection experiments, cells were cultured in 25 cm^2^ tissue culture flasks, at 37 °C, in a 5% CO_2_ atmosphere, for 24 h before infection with *T. gondii* tachyzoites at a ratio of 10:1 (parasites:cell) and monitored by phase contrast microscopy. After various periods of time (60, 120, 240 min, and 24 h after infection), cells were collected and fixed as described below. Non-infected cells were used as controls.

### Fixation, processing, and embedding for light and transmission electron microscopy

The eyes and ARPE-19 cells were fixed in 4% paraformaldehyde, 0.5% glutaraldehyde, and 0.1 mol/l sodium cacodylate buffer (pH 7.4) for 24 h at 4 °C, washed in sodium cacodylate, dehydrated through graded percentages of methanol, and embedded in LRGold (London Resin Co., Reading, UK). Sections (1 µm thick) were stained with 1% toluidine blue in 1% borax solution (TAAB Laboratories, Aldermaston, UK). Eye sections were analyzed on an Axioskop 2-Mot Plus Zeiss microscope (Carl Zeiss, Jena, Germany) for histopathological analysis and quantification of neutrophils and mast cells. Values are shown as mean±standard error (SE) of cells number per mm^2^ of four sections (1 µm) per sample (five samples per animal).

### Immunohistochemical studies

LR Gold-embedded sections (1 µm thick) were incubated with 10% bovine albumin in PBS (PBSA) to block nonspecific binding. Slides were then incubated overnight at 4 °C with polyclonal rabbit anti-ANXA1 antibody (1/200 in 1% PBSA; Zymed, Cambridge, UK). As a control for the reaction, rabbit pre-immune serum (1/200 working dilution; Sigma-Aldrich) was applied in place of the primary antibody. After repeated washings in 1% PBSA, a goat anti-rabbit IgG (Fc fragment-specific) antibody conjugated to 5 nm colloidal gold (1/100; BBInternational, Cardiff, UK) was applied. Silver-enhancing solution (BBInternational) was used to augment gold particle staining. At the end of the reaction, sections were washed thoroughly in distilled water, counterstained with hematoxylin, and mounted in BIOMOUNT (BBInternational). Densitometric analysis for the ANXA1 immunostaining was performed to an arbitrary scale ranging from 0 to 255 using AxioVision software on a Zeiss-Axioskop 2 light microscope, and the data are presented as mean±SEM.

### Ultrastructural immunocytochemistry (transmission electron microscopy)

Ultrathin sections (70 nm) of eye tissues were incubated as follows: (1) in phosphate-buffered solution (PBS) containing 1% egg albumin (PBEA) for 10 min and then (2) in PBS containing 5% egg albumin (PBEA) for 30 min. The sections were then incubated in either polyclonal rabbit anti-ANXA1 (1:200; Zymed) or in normal rabbit serum as a control for 2 h. After washes in PBEA, a goat anti-rabbit IgG antibody (1:100 in PBEA) conjugated to 15 nm colloidal gold (British Biocell, Cardiff, UK) was applied, and after 1 h, sections were washed in PBEA and then in distilled water. Ultrathin sections were stained with uranyl acetate and lead citrate before examination on a ZEISS EM900 electron microscope (Department of Morphology and Genetics, UNIFESP).

Randomly photographed sections of retinal pigment epithelium were used for immunocytochemical analysis. The area of the cell compartment was determined with AxioVision software. The density of immunogold (number of gold particles per µm^2^) was calculated and expressed for each cell compartment. Values are reported as mean±SEM of ten electronmicrographs analyzed per group.

### Statistical analysis

Statistical differences between groups were determined by ANOVA followed, if significant, by the Bonferroni test. In all cases a p-value less than 0.05 was considered as significant.

## Results

### Histopathological analysis of ocular tissues after *T. gondii* infection

Histopathological analysis of the anterior segment of the eye from control and tachyzoites infected animals showed an intense inflammatory response at 24 h after infection, characterized by neutrophil transmigration into the tissues, particularly in the cornea. At 48 and 72 h after infection a decrease in the influx of these cells was noted (Table 1).

**Table 1:** Quantitative analysis of neutrophils in the anterior and posterior segments of the eye.

** **	**Segments of eye**	
**Time/Group**	**Anterior**	**Posterior**
24 h/Control	226±212.2	50±35.6
24 h/Experimental	1327±679.5	115±63.6
48 h/Control	5±5	5±2.6
48 h/Experimental	913.3±471.2**	484±236
72 h/Control	10.33±5.5	3.6±2.7
72 h/Experimental	735±115.8*	4472±1405*

Animals were intravitreally inoculated with 106 tachyzoites in 5 μl of BS (experimental group) or with 5 μl of PBS alone (control group), and the eyes were collected 24, 48,and 72 h after infection. Tissue preparation was performed as described in Methods. Values are displayed as the mean±SEM of tissues analyzed from 4 mice/group. *p<0.01 and **p<0.001 versus the respective control group.

**Figure 1:**
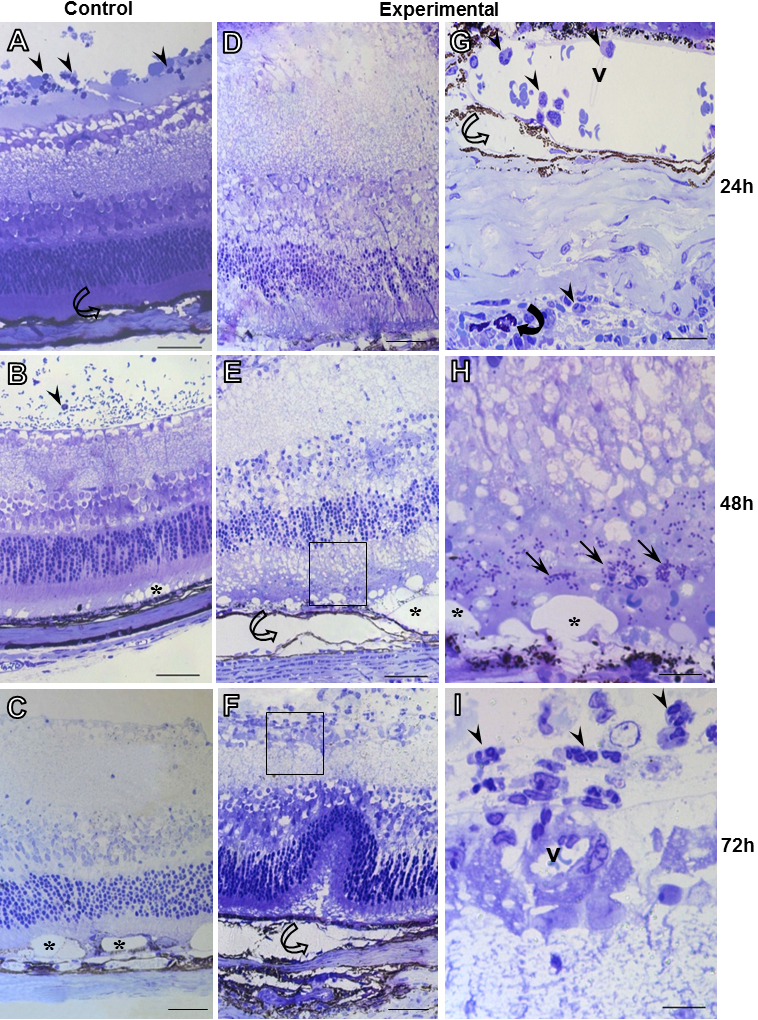
Histopathological analysis of ocular tissues in *T. gondii* infection. Control group (**A**-**C**). Experimental group (**D**-**I**). Disruption of normal retina architecture, formation of gaps (*; **B**, **C**, **E**, and **H**), retinal detachment (hollow curved arrow; **A**, **E**, and **F**) and the presence of inflammatory cells (arrows) in control and experimental groups after 24, 48, and 72 h. Mast cell (filled curved arrow) in sclera region (**G**). Blood vessel (v). Stain: Toluidine blue. Scale bars: 50 μm (**A**-**F**), 10 μm (**G**-**I**).

In the posterior segment of the non-infected (control; Figure 1A-C) and infected eyes (Figure 1D-I), the intravitreal injection caused morphological changes in the retina characterized by its detachment (Figure 1A,E,F) as well as gaps in the outer segments of the photoreceptors (Figure 1B,C,H) and folds of the outer nuclear and plexiform layers directed toward the vitreous chamber (Figure 1F). Despite these non-specific effects, the destruction and disruption of the normal retinal architecture in eyes that were intravitreally inoculated with tachyzoites were more severe compared to control eyes Please spell out the full name the first time mentioned in the text.

At 24, 48, and 72 h post-infection, histopathological analysis of the ocular tissues revealed the presence of inflammatory infiltrates, including in particular neutrophils and mast cells (Figure 1G), and tachyzoites (Figure 1H). These cells exhibited intravascular and extravaseted localization in the choroid, inner ganglionar and plexiform layers, as well as in the humor vitreous (Figure 1I). After 72 h of parasite inoculation, the quantitative analysis of inflammatory cells showed a significant increase in the neutrophil population in the posterior segment, particularly in the retina, as compared to other experimental groups (Table 1).

In addition to neutrophils, mast cells were also observed in the anterior and posterior segments of the eye, including, in particular, the limbus and sclera. Morphological analysis at 24 and 48 h after infection showed a decrease in the mast cell population in the eyes (11.5±0.5 and 12.5±4.8 cells/mm^2^, respectively), as compared to the control animals (23.75±12.29 and 27.2±8.95; p>0.05). In the latter phase of infection (72 h), no difference in mast cell distribution was detected between the control and experimental groups (10±10 and 12.3±3.6 cells/mm^2^, p>0.05, respectively). At this time point, degranulated mast cells were detected in the anterior segments of infected eyes.

### Effect of *T. gondii* infection on ANXA1 expression in the eye

**Figure 2:**
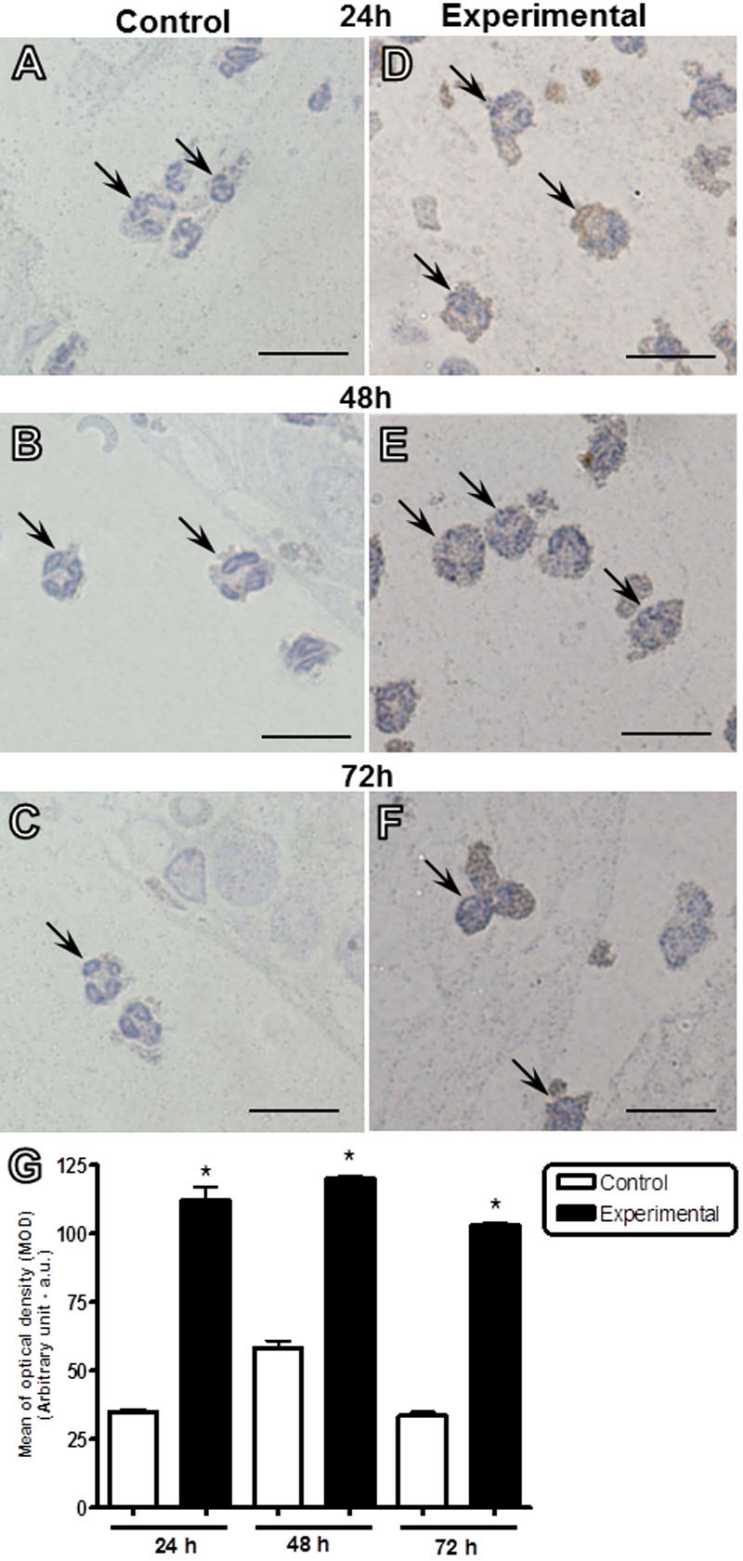
Expression of ANXA1 in neutrophils after intravitreal inoculation of *T. gondii*. Neutrophils (arrows) from infected eyes showed a strong immunoreactivity for ANXA1 in the cytoplasm as compared to control eyes after 24 (**A** and **D**), 48 (**B** and **E**), and 72 h (**C** and **F**). Counterstain: Hematoxylin. Scale bars: 10 µm. **G**: Densitometric analysis of ANXA1 in neutrophils. Values (arbitrary units) are expressed as the mean±SEM of sections analyzed from four mice/group. *p<0.01 versus the respective control group.

ANXA1 expression was analyzed by immunohistochemistry and ultrastructural immunocytochemistry using a polyclonal antibody, which detects the intact (37 kDa) and cleaved (33 kDa) forms of the protein. Immunohistochemical studies showed that neutrophils were immunoreactive for ANXA1 in the ocular tissues of both the control and experimental groups at different time points (24, 48, and 72 h). The neutrophils of parasite-inoculated eyes exhibited a strong immunoreactivity for ANXA1 compared to the neutrophils of control eyes (Figure 2A-F). Densitometric analysis confirmed our histological data, which exhibited a significant increase in the expression of ANXA1 in the neutrophils of *T. gondii*-infected eyes at 24, 48, and 72 h after initial infection as compared to the respective control groups (Figure 2G).

**Figure 3:**
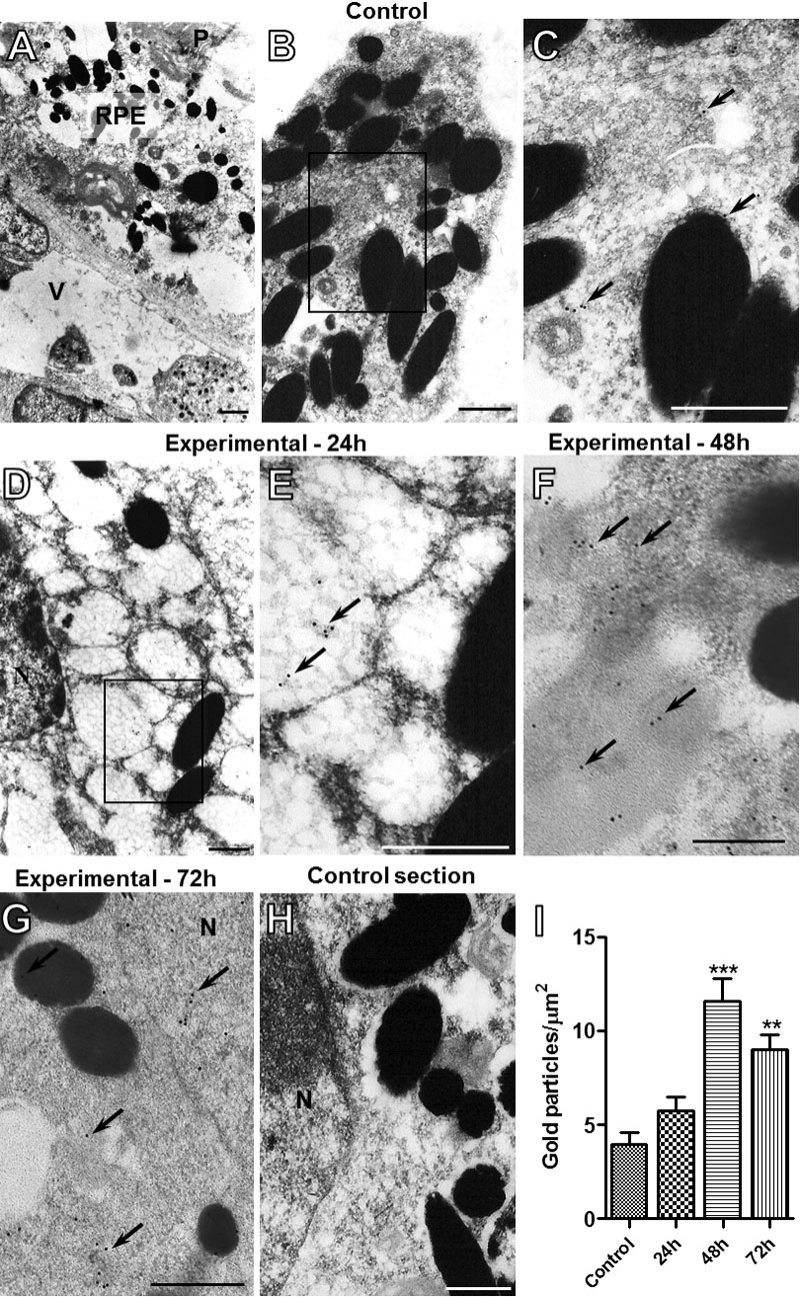
Expression of ANXA1 in the retinal pigment epithelium (RPE) by ultrastructural analysis. Control group (A-**C**). Experimental groups: 24 h (**D** and **E**), 48 h (**F**), and **7**2 h (**G**). **A**: Electron micrograph of RPE localized between a vessel (V) of the choriocapillaris and light-sensitive outer segments of the photoreceptors (P). Marked vacuolization of the RPE (**D**) is observed after intravitreal inoculation of *T. gondii* compared to the control group (**B**). ANXA1 immunogold labeling (arrows) in the cytosol, vacuoles and membrane of pigment granules of RPE of control (**C**) and infected eyes after 24 h (**E**). Also, a significant proportion of ANXA1 immunoreactivity (arrows) observed in infected eyes after 48 (**F**) and 72 h (**G**). No labeling was detected in the control section (**H**). Scale bars: 1 µm (**A** and **D**), 0.5 µm (**B**, **C**, **E**, **F**, **G**, and **H**). Density of ANXA1 immunogold particles in RPE (**I**). Data are mean±SEM of 10 distinct cells analyzed from four mice per group. **p<0.01 and ***p<0.001 versus control group.

Transmission electron microscopy was used to evaluate the expression of ANXA1 in the retinal pigment epithelium (RPE), a tissue that plays an important role in immune defense against *T. gondii* infection. The RPE is located between vessels of the choriocapillaris and light-sensitive outer segments of the photoreceptors (Figure 3A). The posterior segment of the eye was significantly affected by *T. gondii* infection after 24, 48, and 72 h, as previously described, and exhibited a marked vacuolization of RPE cells (Figure 3D) as compared to the control group (Figure 3B). ANXA1 immunogold labeling was detected in the RPE of control and infected eyes, with localization in the cytosol, vacuoles and membrane of pigment granules (Figure 3C,E,F,G). *T. gondii* infection produced in the RPE a significant increase of ANXA1 immunoreactivity in their subcellular compartments (plasma membrane, cytoplasm and nucleus) at 48 and 72 h (Figure 3F,G), compared to control group, that was confirmed by quantification of gold particles (Figure 3I). No labeling was detected in the control section (Figure 3H).

### Analysis of ANXA1 expression in human retinal pigment epithelial cells (ARPE-19) after *T. gondii* in vitro infection

Upon detection of ANXA1 in the RPE, we performed in vitro studies using ARPE-19 cells to investigate the modulation of ANXA1 expression after *T. gondii* infection.

**Figure 4:**
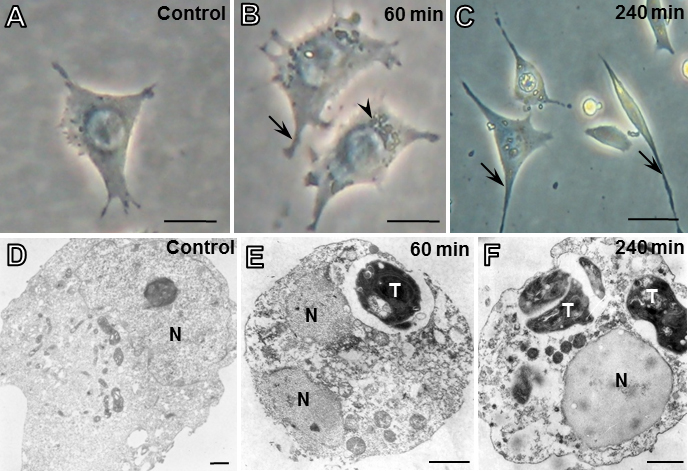
Analysis of ARPE-19 cells after *T. gondii* infection by light and transmission electron microscopy. Non-infected (control; **A**) and infected cells after 60 (**B**) and 240 min (**C**) with thin and long extensions (arrows). Intracellular parasites (arrowhead). Electron micrograph of control ARPE-19 cells (**D**) and infected with parasitophorous vacuoles containing variable numbers of *T. gondii* tachyzoites (T) after 60 (**E**) and 240 min (**F**). N, nucleus. Scale bars: 25 µm (**A**), 10 µm (**B**, **C**), 1 µm (**D**-**F**).

Initially, ARPE-19 cells were analyzed by phase contrast microscopy during control conditions that allow for the retention of features characteristic of the RPE, including well defined cell borders and noticeable pigmentation. After *T. gondii* infection, ARPE-19 cells exhibited morphological alterations, including cytoplasmic retraction and the formation of long and thin extensions with increased presence of intracellular parasites, particularly 60 and 240 min after initial infection (Figure 4A-C). The morphological and structural alterations associated with *T. gondii* infection were confirmed by transmission electron microscopy analysis. As shown in Figure 4D-F, ARPE-19 cells exhibited well defined parasitophorous vacuoles containing variable numbers of *T. gondii* tachyzoites 60 and 240 min after initial infection.

**Figure 5:**
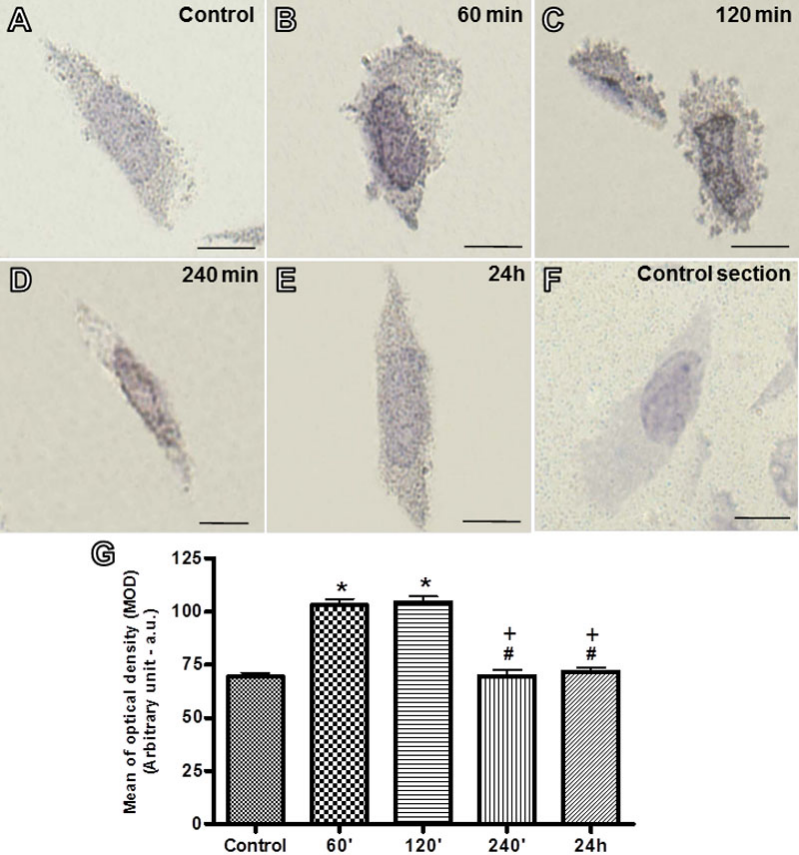
Immunohistochemistry for ANXA1 in ARPE-19 cells. ANXA1 immunostaining was detected in the nucleus and cytoplasm of ARPE-19 cells (**A**-**E**). After 60 and 120 min of infection, an intense immunoreactivity of ANXA1 was detected in the infected cells (**B** and **C**) as compared to control cells (**A**). In the later time points of infection (240 min and 24 h), decreased expression of ANXA1 was noted (**D** and **E**). Absence of ANXA1 immunostaining in ARPE-19 cells incubated with pre-immune serum (**F**). Counterstain: Hematoxylin. Bars: 10 µm. **G**: Densitometric analysis of ANXA1. Values (arbitrary units) are expressed as the mean±SEM of sections analyzed from 10 cells/ group. *p<0.001 versus the control group; ^#^p<0.001 versus 60 min; ^+^p<0.001 versus 120 min.

Immunohistochemistry studies showed ANXA1 localization in the nucleus and cytoplasm of ARPE-19 cells. At 60 and 120 min after initial infection with *T. gondii*, an intense immunoreactivity of ANXA1 was detected in the nucleus and cytoplasm of infected cells compared to control cells (Figure 5A-C). At 240 min and 24 h after infection, a low level of ANXA1 expression was noted in these cells (Figure 5D,E). No labeling was detected in ARPE-19 cells incubated with rabbit pre-immune serum (Figure 5F). Densitometric analysis revealed that *T. gondii* infection significantly augmented ANXA1 protein levels at 60 and 120 min after infection, followed by a decrease at 240 min and 24 h after infection (Figure 5G).

## Discussion

In this study we have investigated the ocular inflammatory response and the expression pattern of anti-inflammatory protein, ANXA1, in inflammatory and retinal pigment epithelial (RPE) cells in in vivo and in vitro models of *T. gondii* infection (RH strain).

Histopathological studies showed that the intravitreal inoculation of tachyzoites in mice provoked an intense influx of inflammatory cells, especially neutrophils, in anterior (24 h) and posterior (72 h) eye segments with consequent destruction and disruption of the normal retinal architecture and RPE vacuolization as compared to the control groups. Similar observations were previously reported for mice that were intravitreally inoculated with another strain of *T. gondii* (ME-49) [23], supporting the use of C57BL/6 mouse as a reproducible and analogous experimental model for studying human ocular toxoplasmosis [25,26].

Our laboratory previously found that eyes infected with *T. gondii* (RH strain) presented visible signs of mast cell degranulation associated with inflammatory response [27]. Mast cells can release an array of potent mediators, which can induce both rapid and delayed inflammatory response, thereby promoting leukocyte recruitment, contributing to development of eye diseases, including conjunctivitis, atopic keratoconjunctivitis and uveitis [28-30]. Our data revealed that the presence of parasites at 24, 48, and 72 h after initial infection did not alter mast cell numbers as compared to in the control group, although at 72 h after infection all mast cells were degranulated in the anterior eye segment. Previous rodent models of *T. gondii* (RH strain), intraperitonial infection found degranulation and cytoplasmic vacuoles containing tachyzoites in peritoneal mast cells, suggesting their involvement in the immune response to *T. gondii* infection [31] through the release of potent mediators, such as leukotriene B4 (LTB_4_) [32].

The accumulation and subsequent activation of leukocytes are central events in the inflammatory response. Thus, the further elucidation of the molecular mechanisms regulating leukocyte influx into inflamed tissues may provide a better understanding of the etiology of inflammatory diseases, as well as important targets for anti-inflammatory therapy [33]. The term “anti-inflammation” has been used to describe the balance between pro and anti-inflammatory mediators that work together to initiate, maintain, and finally resolve inflammatory reactions. ANXA1, a protein of 37 kDa, was originally identified as a mediator for several anti-inflammatory actions of glucocorticoids [10]. Pharmacological studies with ANXA1 demonstrated its effects on inflammatory cells, including mast cells [17,18], neutrophils [14] and eosinophils [34]. Furthermore, the development of knockout mice for ANXA1 [35] and ultrastructural immunocytochemical analysis [14,36] have allowed for the better definition of roles played by endogenous ANXA1 in several cellular functions, including phagocytosis, migration, and synthesis of mediators [37,38].

Despite the extensive literature on the pharmacological effects of ANXA1 and its mimetic peptides in the specific responses in cells using in vitro and in vivo procedures [15,39,40], few studies have linked this protein to intraocular inflammation.

Immunohistochemical studies in the ocular tissues revealed the expression of ANXA1 in the neutrophils, especially its localization in the cytoplasm. At 24, 48, and 72 h after intravitreal tachyzoite infection, a significant increase in ANXA1 levels in the neutrophils of the anterior and posterior eye segment was detected compared to control cells. These data showed a modulation of the endogenous ANXA1 in the cells that transmigrated to the inflammatory sites, suggesting a role for ANXA1 in the process of neutrophil activation in this experimental infection model. In a model of intraocular inflammation induced by carrageenin administration in the rat paw, an increase in the endogenous levels of ANXA1 in the neutrophils was associated with a decrease in the migration of these cells in the latter phase of the inflammation, suggesting a role for this protein in the control of neutrophil activation and consequent migration and contribution to the resolution of the inflammatory response [41]. Several studies have observed this anti-migratory effect of ANXA1 [42]. Immunocytochemical studies and in situ hybridization showed that the expression of endogenous ANXA1 increased in the plasma membrane of circulating neutrophils during the inflammatory process and after transmigration in the cytoplasm [14]. These data indicate that the protein ANXA1 regulates the leukocyte transmigration and the production of proinflammatory mediators in neutrophils. Recent investigation has also showed that in ANXA1 null mice, the inflammatory process induced by zymosan exacerbated leukocyte transmigration in the peritoneal cavity [22].

The changes in RPE and the segments of photoreceptors represent important clinical aspects of ocular toxoplasmosis, resulting in retinochoroiditis. Further, the destruction of the retina in the experimental ocular toxoplasmosis model, where intravitreal and i.p. routes were used to administer *T. gondii* (ME-49 strain) inoculation, was associated with the migration of RPE cells proximal to the parasites in this tissue, suggesting a role of RPE in the immune defense against infection [43].

Our ultrastructural immunocytochemistry data revealed an overexpression of the anti-inflammatory ANXA1 protein in RPE cells after 48 and 72 h infection by *T. gondii*. Based on the relevance of functional characteristics of human RPE cells to the inflammatory events [44], and due to the possible compare and confirm the data obtained on rodent model in vivo, we also monitored the expression of ANXA1 in the human RPE (ARPE-19 cells) in experimental conditions. Interaction of these cells with parasites induced ANXA1 expression, with a significant increase in its level after 60 and 120 min, followed by a decrease at later times (240 min and 24 h). Most studies investigating the effect of ANXA1 on epithelial cells have identified an antiproliferative role [37] and few of them discuss this role during inflammatory processes. One such investigation showed increased promoter activity of the *ANXA1* gene in lung epithelial cells at 6 h after induction of endotoxemia in mice. This activation was associated with the activity of these cells in controlling local and systemic responses to LPS administration [21]. In chronic inflammation, the expression of ANXA1 is also positively modulated in human epithelial cells in nasal polyps cases after treatment with glucocorticoids, suggesting an inhibitory role of ANXA1 in epithelial proliferation, which is common to this pathology [19]. Thus, the increased levels of ANXA1 in the ARPE-19 cells are likely for cellular activation and the phagocytosis of the parasites, indicating an immune protective role in the tissue.

In conclusion, our results demonstrated that intravitreal infection of *T. gondii* in a murine model provoked an intense inflammatory response characterized by leukocyte accumulation in the ocular tissues and morphological changes that are clinically significant in the retina after 72 h of infection. Further, by immunohistochemical analyses, we showed for the first time, that the experimental infection by *T.gondii* positively modulates the expression of ANXA1 in the neutrophils and RPE cells involved in the intraocular inflammatory responses, which may constitute an important target for therapy studies in ocular toxoplasmosis.
